# Crusted Scabies in an Elderly Patient

**DOI:** 10.4269/ajtmh.23-0202

**Published:** 2023-09-25

**Authors:** Ying Yang, Hong Shen, Ze-Hu Liu

**Affiliations:** ^1^ Department of Intensive Care Unit, Hangzhou Third People’s Hospital, Affiliated Hangzhou Dermatology Hospital, Zhejiang University School of Medicine, Hangzhou, China;; ^2^ Department of Dermatology, Hangzhou Third People’s Hospital, Affiliated Hangzhou Dermatology Hospital, Zhejiang University School of Medicine, Hangzhou, China

A 92-year-old woman who had progressive dementia for about a year was transferred from a long-term care facility to the intensive care unit (ICU) in May 2014. The transfer was meant to address dyspnea, fever, and confusion, which was secondary to methicillin-resistant *Staphylococcus aureus* bacteremia. The patient had a month’s history of generalized, pruritic, and erythematous skin rash. A week after transfer to the ICU, bacteremia was controlled using intravenous antibiotics, whereas seven healthcare workers complained of nocturnal pruritus and itchy lesions. Three of the patient’s family members also admitted pruritus for more than 1 month. Hyperkeratotic yellow-crusted lesions accompanied with significant edema were detected on both hands (Figures 1 and 2). Eosinophilia (2.75 × 10^9^/L; normal: < 0.5 × 10^9^/L) was also noted. The serum Immunoglobulin E (IgE) level was more than 2,500 IU/mL (normal: < 100 IU/mL). Skin scrapings confirmed the presence of *Sarcoptes scabiei* adult mites (Figure 3), and the patient was diagnosed with crusted scabies. A local outbreak of scabies was reported, and control measures were immediately instituted. All healthcare workers underwent a screening dermatological examination. Classic scabies was diagnosed in seven healthcare workers, five nurses, two inpatients, and family members. The patient was successfully treated with combination therapy including keratolytic solution (Chinese traditional medicine) and topical scabicides (5% precipitated sulfur petrolatum) (Figures 4 and 5). The local scabies outbreak completely resolved within a month.

**Figure 1.**
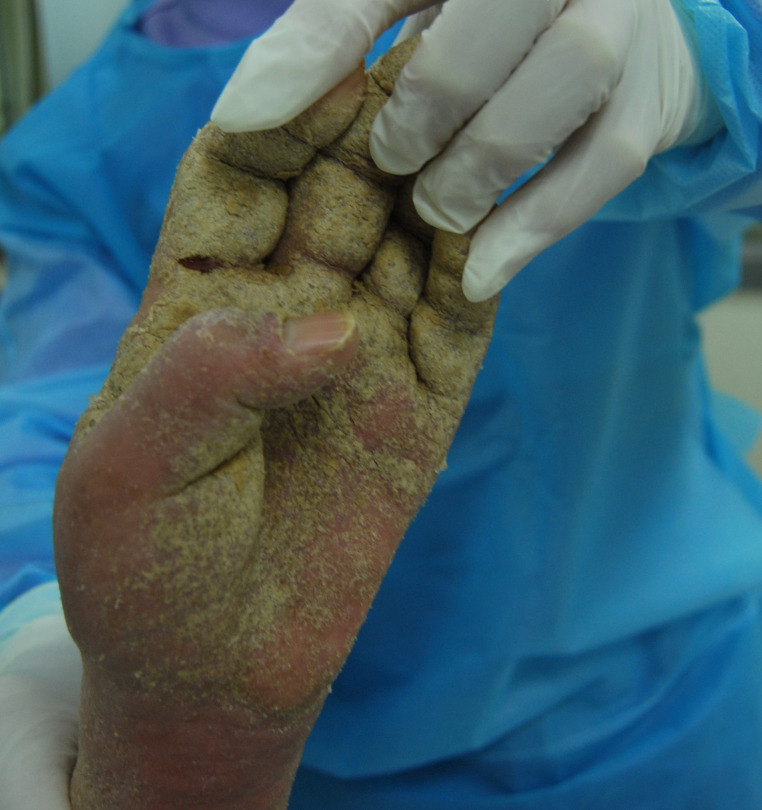
Hyperkeratotic yellow-crusted lesions on the right hand.

**Figure 2.**
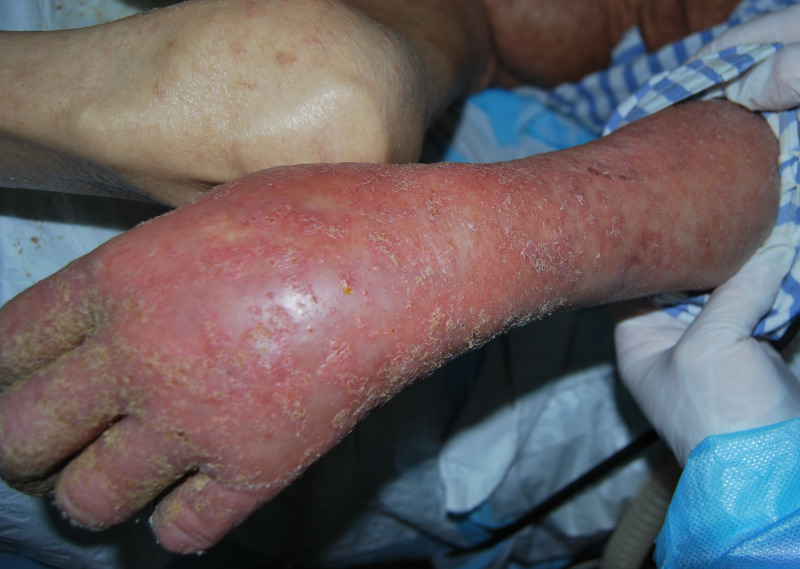
Yellow-crusted lesions with significant edema on the left hand.

**Figure 3.**
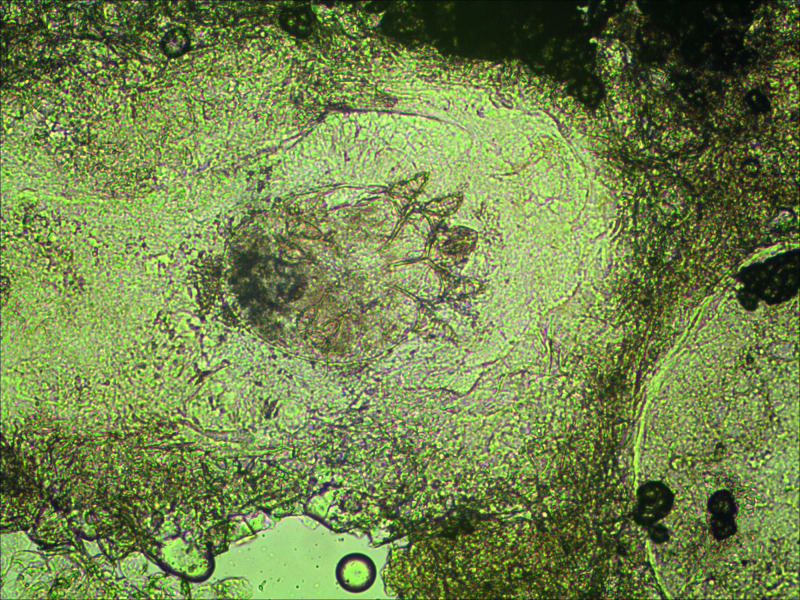
Presence of *Sarcoptes scabiei* adult mites under direct microscopy.

**Figure 4.**
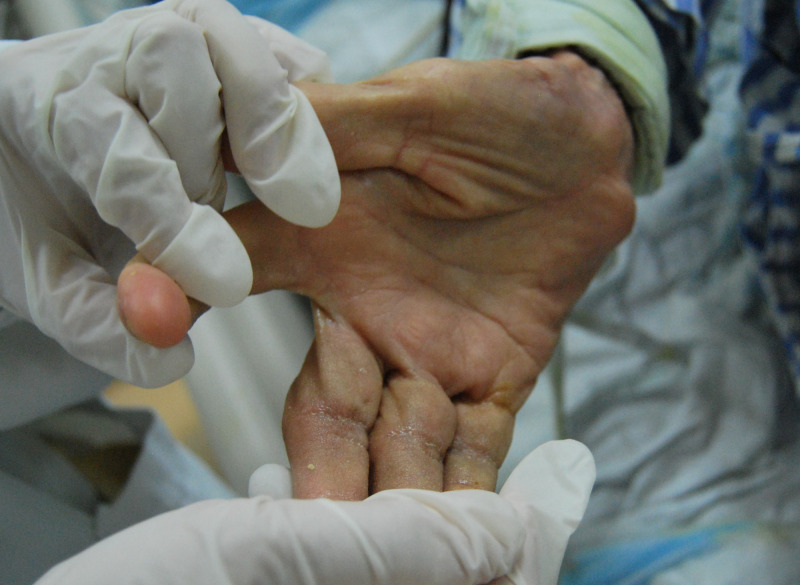
Complete resolution in the right hand.

**Figure 5.**
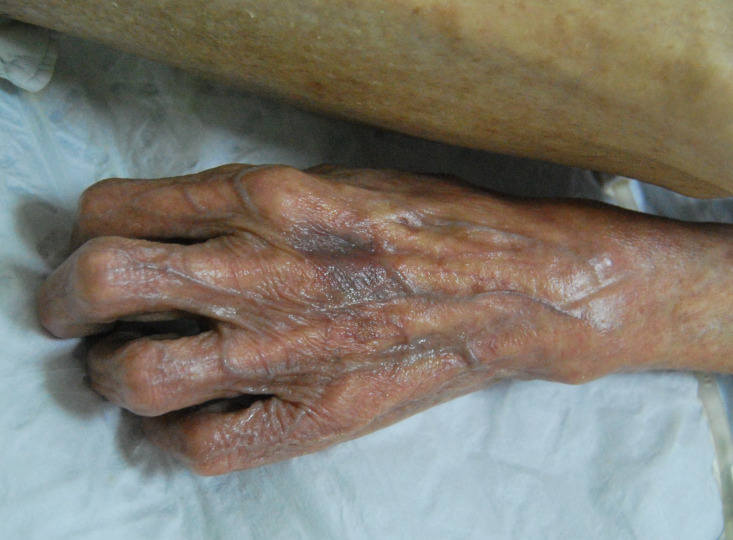
Complete resolution in the left hand.

Crusted scabies, also known as Norwegian scabies, was first described by Danielson and Bock in 1848.^1^ It is thought to be the result of inadequate host response to the *S. scabiei* mite. Crusted scabies is mostly found in immunocompromised, malnourished, and disabled individuals. Eosinophilia and elevated IgE levels are observed more often in patients with crusted scabies than in those with the noncrusted form.^2^ Crusted scabies is highly contagious, considering the host may carry more than a million mites and may shed thousands of them daily. This explains the numerous large outbreaks that have been experienced in nursing facilities.^3^ Crusted scabies was identified as a core transmitter in scabies epidemic cycles.

Classic cases can be treated topically with permethrin, lindane, or crotamiton. Severe cases of crusted scabies are usually resistant to topical scabicidal treatment, which is why the administration of oral ivermectin has been adopted, especially in developed countries. Despite the unavailability of ivermectin in developing countries, including China, this patient was successfully treated with a combination therapy of keratolytic solution and topical precipitated sulfur petrolatum.

Clinically, crusted scabies presents as psoriasiform dermatitis with an acral distribution. Although crusted scabies is atypical scabies, the yellow-crusted plaque is highly characteristic of crusted scabies. Generally, the long-term care facility staff did not recognize the characteristic skin lesions even long after admission. The failure of staff to diagnose scabies in patients upon admission may be attributable to mild pruritus or a lack of pruritus in these mentally challenged patients.^4^ Clinical diagnosis should be suspected once family members report itching incidences or history, which can be aided by direct microscopy examination. The tape method is simple and useful for the diagnosis of crusted scabies, as it is less sensitive than traditional scraping.^5^ In our opinion, scraping from the crusts using saline is also very sensitive. Early diagnosis of crusted scabies may prevent the possibility of large outbreaks, which are further associated with prolonged hospitalization, possible ward closure, and economic burdens.

## Financial Disclosure

This work was supported by the Hangzhou Science and Technology Bureau, China (Grant no. 202004A17).
